# Methyl-Acetandilide

**Published:** 1890-03-22

**Authors:** 


### METHYL-ACETANILIDE.

Dr. Thomas Fraser, Professor of Materia Medica in the University of Edinburgh, publishes a lecture on "the analgesic action of methyl-acentanilide or exalgine." Methyl-acetanilide or exalgin, is one of the four methyl derivatives of acetanilide, discovered by Hoffman, and its formula is $C_9H_{11}NO$. It occurs in the shape of colourless needle-shaped crystals of considerable length, which have a faintly aromatic odour, and a slightly pungent taste. It is nearly insoluble in water, but freely soluble in dilute alcohol. From 16 to 20 grains may be dissolved in half a drachm of rectified spirit, and this diluted with three or four ounces of water. A tea-spoonful containing from half a grain to a grain may be used. Dr. Fraser has administered the preparation for neuralgia, and for pain in various diseases. The largest quantity given in twenty-four hours was fourteen grains, from which no disagreeable effects resulted. Cases are detailed of neuralgia of the right inferior and superior trochlear nerves, of sciatica, of herpes cervico-brachialis, of neuralgia of the right arm, of toothache, of locomotor-ataxy, in all of which pain was relieved. Out of eighty-eight cases, sixty-seven experienced relief from pain. The best results were obtained in neuralgias. In conclusion Dr. Fraser observes, "the results justify the hope, that exalgine may take a useful and important place among the remedies by which pain is relieved, and it has the enormous advantage of being free from the disturbances and inconveniences that are associated with the action of nearly all other subduing agents, and from the dangers inseparable from the use of the more powerful of these agents." Now it is only quite recently—within the last few months—that the action of methyl-acetanilide has been made known by Bardet, Binet, Dujardin-Beaumetz, and others, and Dr. Fraser seems to have tried it first in this country. As a rule, we look with some degree of doubt on new remedies; for it is so often noticeable that a new remedy is spoken of with great approval, soon, however, lapsing into the mass of utterly forgotten preparations. Dr. Fraser's observations are, however, so careful and complete, that we imagine a better fate is reserved for methyl-acetanilide. In the "Retrospect" of February 8th, is a notice of acetanilide and its power of reducing febrile temperature; and some time back we remarked on the occasionally evil results arising from the use of antifebrin, antipyrin, &c. In connection with this subject we again quote Dr. Fraser, who writes, "the study of members of this group has shown that very slight changes in their chemical composition, may be followed by marked changes in their pharmacological action." Methyl-acetanilide illustrates this, for it is found to be a more powerful analgesic than acetanilide. When unpleasant results arise from the use of these anti-febrile drugs, much must be attributed to peculiar constitution or temperament, or idiosyncracy. But there is another danger of the drugs not acting as it is intended they should, which, as Dr. Fraser points out, may depend on chemical preparation. This, however, is a danger the chance of which must be incurred. For, as observed in the "Retrospect," February 15th, under the heading of "Adulteration of Drugs," "irrespective of intentional adulteration, of which we believe there is little, drugs used in composition differ in strength themselves, and a very slight accidental variation of chemical process may produce a different result . . . the greatest care cannot always secure exactly similar results." This is a matter which the chemists must look to.

